# Supplementary material for: High Nitric Oxide Concentration Inhibits Photosynthetic Pigment Biosynthesis by Promoting the Degradation of Transcription Factor HY5 in Tomato
Source: Int J Mol Sci. 2022 May 27;23(11):6027. doi: 10.3390/ijms23116027 (PMC9181159; doi:10.3390/ijms23116027)
Supplement: Supplementary file 1 [file ijms-23-06027-s001.zip › Supporting Information.pdf]

Supporting Information

Article title: High Nitric Oxide Concentration Inhibits Photosynthetic Pigment Biosynthesis by Promoting the Degradation of Transcription Factor HY5 in Tomato

Authors: Lingyu Wang[1], Rui Lin[1], Jin Xu[1], Jianing Song[1], Shujun Shao[1], Jingquan Yu[1,2] and Yanhong Zhou[1,2,*]

The following Supporting Information is available for this article:

Figure S1. The *gsnor* mutant transgenic tomato plants.

Figure S2. Relative expression of *POR*s and *PSY*s.

Figure S3. Both endogenous and exogenous NO inhibit the photosynthetic capacity.

Figure S4. NO signalling regulates pigments production in a dose-dependent manner.

Figure S5. The *hy5* mutant transgenic tomato plants.

Figure S6. HY5 binding motif in the promoters of *PORC* and *PSY2* in tomato.

Figure S7. HY5 directly binds to the *PORC* and *PSY2* promoters and activites their transcription.

Figure S8. Efficiency of *GSNOR* silencing by virus-induced gene silencing (VIGS) in WT and *hy5*.

Figure S9. NO donors inhibit the expression of photosynthetic pigments biosynthesis-related genes through HY5.

Table S1. Primers used for RT-qPCR.

Table S2. Primers used for DNA constructs.

Table S3. Primers used for EMSA.

Table S4. Primers used for ChIP-qPCR.

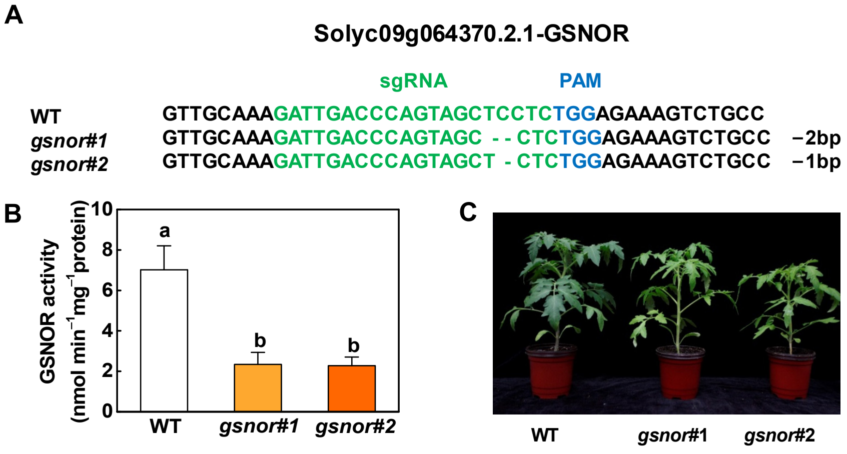

Figure S1. The *gsnor* mutant transgenic tomato plants. (**A**) Schematic illustration of the sgRNA in wild type (WT) and two alleles of *gsnor* from CRISPR/Cas9 T2 mutant lines. Green font presents the sgRNA target sequence, and blue font presents protospacer-adjacent motif (PAM) sequence. The *gsnor#1* mutant carries a 2-bp deletion in the GSNOR ORF, and the *gsnor#2* mutant carries an 1-bp deletion in the GSNOR ORF, which leads to a frame shift and the generation of a premature stop codon, TGA. (**B**) GSNOR activity in WT, *gsnor#1*, and *gsnor#2*. Data are shown as means ± SD (n= 4). Different letters indicate significant difference at $p < 0.05$ according to Tukey's test. (**C**) Phenotypes displayed by WT, *gsnor#1*, and *gsnor#2* mutants.

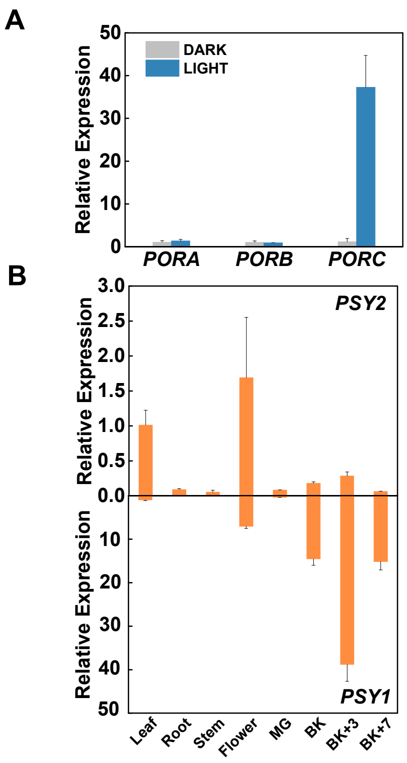

Figure S2. Relative expression of *POR*s and *PSY*s. (**A**) Relative transcripts of *PORA*, *PORB*, and *PORC* in leaves of WT under dark or 6h light conditions. (**B**) Relative transcripts of *PSY1* and *PSY2* in different tissues and the mature green (MG), breaker (BK), three days after breaker (BK+3), and seven days after breaker (BK+7) ripening stages. Data are shown as means ± SD (n = 3).

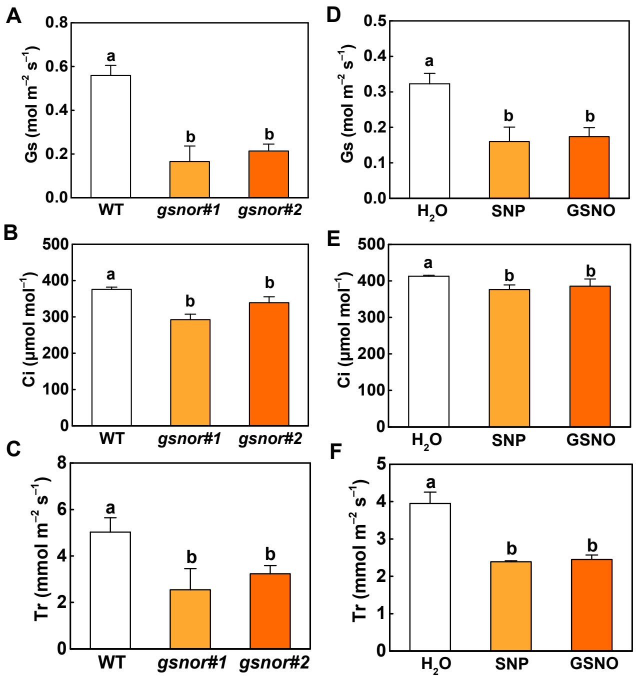

Figure S3. Both endogenous and exogenous NO inhibit the photosynthetic capacity. (**A–C**) Internal CO_2 concentration (Ci), transpiration rate (Tr), and stomatal conductance (Gs) in WT, *gsnor#1*, and *gsnor#2*. (**D–F**) Ci, Tr, and Gs in tomato plants treated with H_2O, 500 μM SNP, and 500 μM GSNO. Data are shown as means ± SD (n = 3). Different letters indicate significant difference at $p < 0.05$ according to Tukey's test.

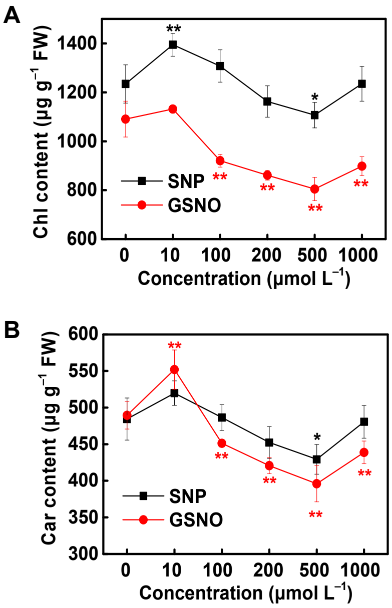

Figure S4. NO signalling regulates pigments production in a dose-dependent manner. (**A**,**B**) Chlorophyll and carotenoid content of leaves were treated with various concentration gradient (10, 100, 200, 500, and 1000 μM) of sodium nitroprusside (SNP) and S-nitroso glutathione (GSNO) as foliar spray. Water was represented by 0 μM of spraying treatment. Data are shown as means ± SD (n = 4). Statistically significant differences were indicated using asterisks (**, $p < 0.01$; *, $p < 0.05$), according to Tukey's test.

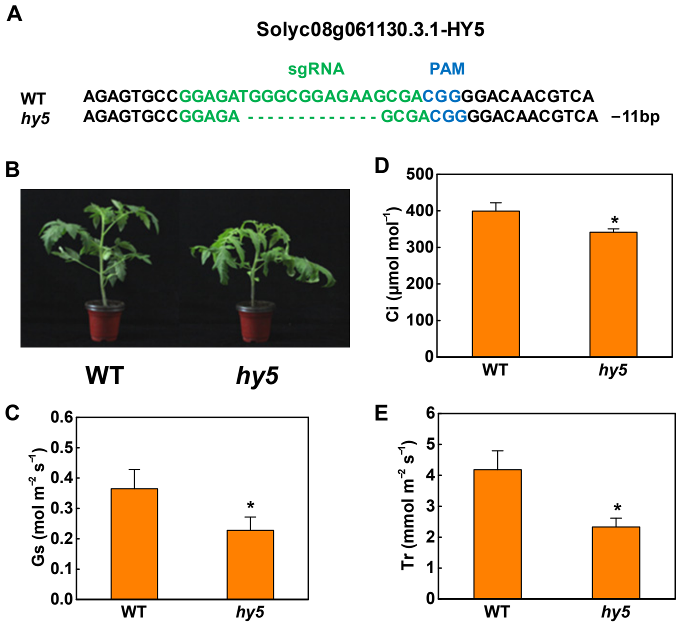

Figure S5. The *hy5* mutant transgenic tomato plants. (**A**) Schematic illustration of the sgRNA in wild type (WT) and the CRISPR/Cas9 T2 *hy5* mutant. Green font presents the sgRNA target sequence, and blue font presents protospacer-adjacent motif (PAM) sequence. The *hy5* mutant carries a 11-bp deletion in the HY5 ORF, which leads to a frame shift and the generation of a premature stop codon, TAG. (**B**) Phenotypes displayed by the WT and *hy5* mutant plants. (**C–E**) Ci, Tr, and Gs in WT and *hy5*. Data are shown as means ± SD (n = 4). Statistically significant differences were indicated using asterisks (*, $p < 0.05$), according to Tukey's test.

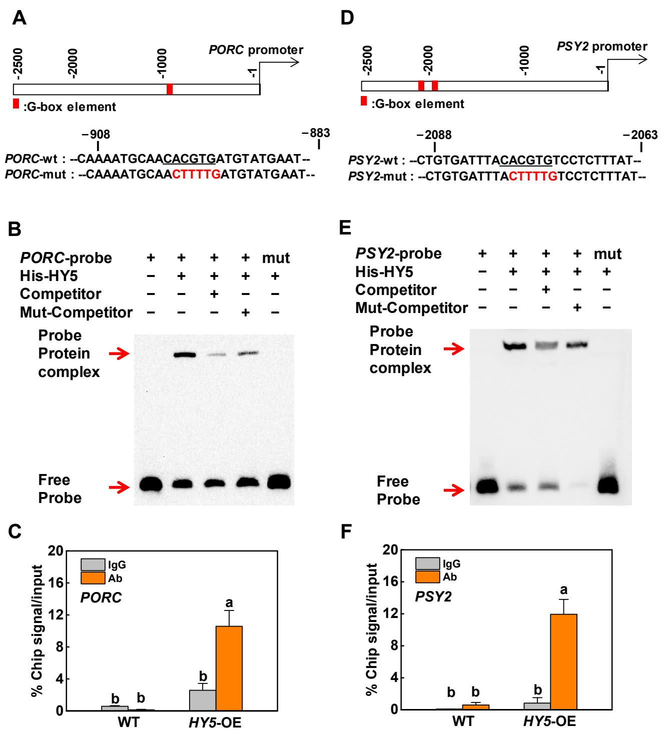

Figure S6. HY5 directly binds to the *PORC* and *PSY2* promoters and activites their transcription. (**A**,**D**) HY5 binding motif in the promoters of *PORC* and *PSY2* in tomato. The wild-type (*PORC*-wt, *PSY2*-wt) and mutant (*PORC*-mut, *PSY2*-mut) oligos were used in EMSA assays. (**B**,**E**) EMSA assay. The His-HY5 recombinant protein was incubated with biotin-labeled wild-type or mutant *PORC* and *PSY2* oligos. The protein purified from the empty vector was used as a negative control. (**C**,**F**) ChIP-qPCR assay. Leave samples from WT and *HY5*-OE tomato plants were precipitated with an anti-HA antibody. A control reaction was processed simultaneously using mouse IgG. The ChIP results are presented as percentages of the input DNA. Data are shown as means ± SD (n = 3). Different letters indicate significant difference at $p < 0.05$ according to Tukey's test.

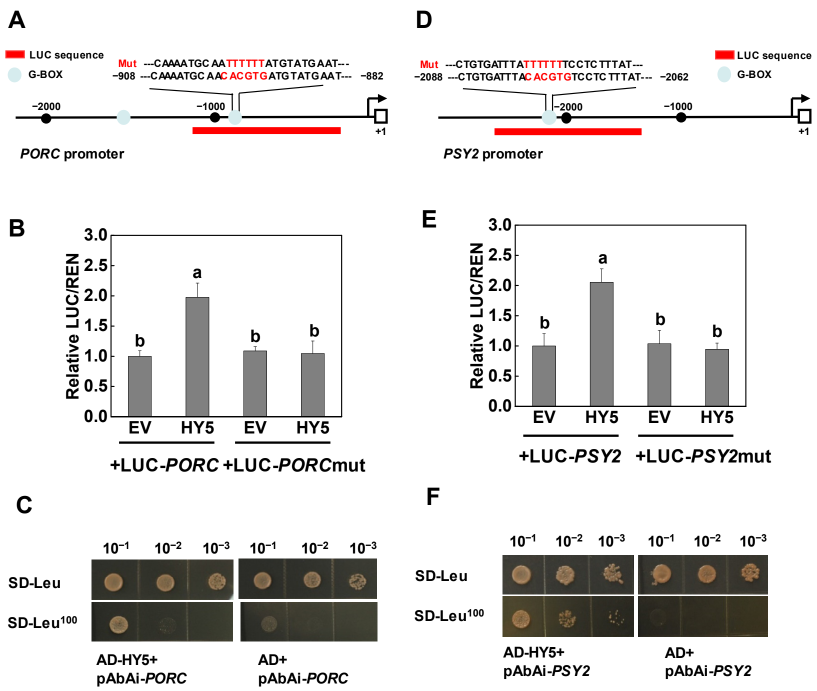

Figure S7. HY5 directly binds to the *PORC* and *PSY2* promoters and activites their transcription. (**A**,**D**) Promoter fragments of *PORC* (–1186 to –1) and *PSY2* (–2267 to –1699) used for the dual luciferase assay. (**B**,**E**) Relative LUC/REN ratio. Tobacco (*Nicotiana benthamiana*) leaves were infiltrated, and the firefly LUC and REN LUC were assayed 3 days after infiltration. The ratio of LUC/REN of the empty vector (EV) plus *PORC* or *PSY2* promoter was set as 1. Data are shown as means ± SD (n = 6). Different letters indicate significant difference at p < 0.05 according to Tukey's test. (**C**,**F**) Yeast-one hybrid assay. The reporter vector was introduced into yeast strain Y1HGold with the effector vector. The transformants were grown on a selective medium (SD/-Leu) without or with 100 ng ml^{-1} AbA. A combination of reporter vector and the empty vector pGADT7 was used as a negative control.

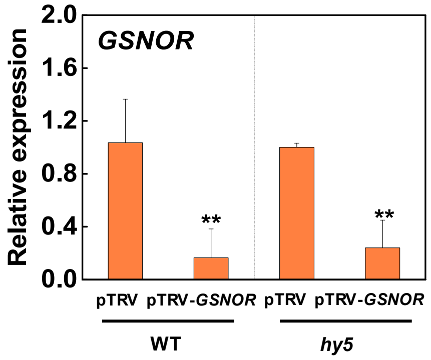

Figure S8. Efficiency of *GSNOR* silencing by virus-induced gene silencing (VIGS) in WT and *hy5*. Statistically significant differences were indicated using asterisks (**, $p < 0.01$), according to Tukey's test.

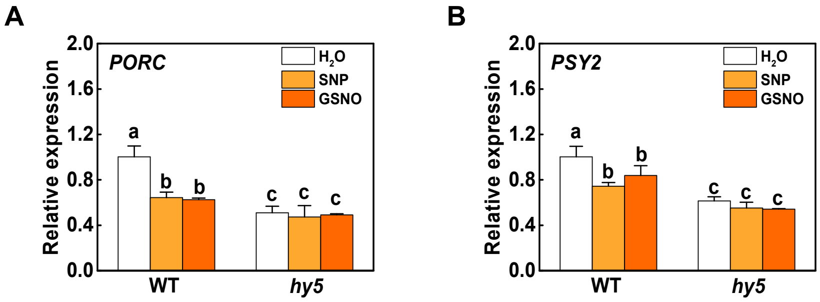

Figure S9. NO donors inhibit the expression of photosynthetic pigments biosynthesis-related genes through HY5. (**A**) Relative transcripts of *PORC* and (**B**) *PSY2* in WT and *hy5* plants treated with H_2O, 500 μM SNP, and 500 μM GSNO. Data are shown as means ± SD (n = 3). Different letters indicate significant difference at $p < 0.05$ according to Tukey's test.

Table S1. Primers used for RT-qPCR.

ID	Primer sequence(5′-3′)
qPCR-*ACTIN*-F	GTCCTCTTCCAGCCATCCAT
qPCR-*ACTIN*-R	ACCACTGAGCACAATGTTACCG
qPCR-*PSY1*-F	CTGGAAGGGTGACCGATAAA
qPCR-*PSY1*-R	ACCAAAGATGCCCATACAGG
qPCR-*PSY2*-F	CCGAATTCCGAGGTCTCATA
qPCR-*PSY2*-R	AAAATTCCACCCCTGTCTCC
qPCR-*PORA*-F	ACTCCTGCAATCACCCAGTC
qPCR-*PORA*-R	TCGCCTATAGCCTTTGCTGT
qPCR-*PORB*-F	CATCTCCCGGTGTAACGAGT
qPCR-*PORB*-R	ACATGCCATTTCCCTGTCTC
qPCR-*PORC*-F	CTCCAAGGGCAAAGCTAGTG
qPCR-*PORC*-R	GCTCTTACAACACCGCCATT
qPCR-*HY5*-F	GCAAGCGACGAGTTCTAT
qPCR-*HY5*-R	ATCTCCGGCACTCTTCTG

Table S2. Primers used for DNA constructs.

ID	Primer sequence(5′-3′)
pET32a-HY5-BamHI-F	gccatggctgatatcggatccATGCAAGAGCAAGCGACG
pET32a-HY5-HindIII-R	gtggtggtggtggtgctcgagCTTCCTCCCTTCCTGTGC
SK-HY5-BamHI-F	cgctctagaactagtggatccGAATGCAAGAGCAAGCGACG
SK-HY5-KpnI-R	tgatttcagcgaattggtaccCTTCCTCCCTTCCTGTGCA
LUC-*PORC*-XhoI-F	ggtaccgggccccccctcgagACCTCGAAGTCTAGCAGCCTAAAT
LUC-*PORC*-BamHI-R	cgctctagaactagtggatccTAAGAAGTTCAATTTTGGGAATTTTT
LUC-*PSY2*-XhoI-F	ggtaccgggccccccctcgagAGTTTTTACGTGGTCCGCTCTT
LUC-*PSY2*-BamHI-R	cgctctagaactagtggatccAGCTAACTAGCTTCCGAGGGAAT
pTRV-*GSNOR*-XbaI-F	tgctctagaAGCAACCCATTCAGCAAGTC
pTRV-*GSNOR*-BamHI-R	cgcggatccTGTTTATGTCCGCAAGTGTC
AD-HY5-NedI-F	ggaattccatatgATGCAAGAGCAAGCGACGA
AD-HY5-BamHI-R	cgggatccCTTCCTCCCTTCCTGTGC
pAbAi-*PORC*--F	cttgaattcgagctcggtaccGGTTGATGATCTTGAGTTATTTTTCG
pAbAi-*PORC*--R	atacagagcacatgcctcgagCGAGAACCAGTAATTTATGACTTGTT T
pAbAi-*PSY2*-KpnI-F	cttgaattcgagctcggtaccAGGTTCGTACGTTCTGGTGTATGA
pAbAi-*PSY2*-XhoI-R	atacagagcacatgcctcgagAGATAGTCAATAATTTCTTGATTATCA ATAAAA

Table S3. Primers used for EMSA.

ID	Primer sequence(5′-3′)
PORC-wt-F	CAAAATGCAACACGTGATGTATGAAT
PORC-wt-R	ATTCATACATCACGTGTTGCATTTTG
PORC-mut-F	CAAAATGCAACTTTTGATGTATGAAT
PORC-mut-R	ATTCATACATCAAAAGTTGCATTTTG
PSY2-wt-F	CTGTGATTTACACGTGTCCTCTTTAT
PSY2-wt-R	ATAAAGAGGACACGTGTAAATCACAG
PSY2-mut-F	CTGTGATTTATCCTCTTTAT
PSY2-mut-R	ATAAAGAGGATAAATCACAG

Table S4. Primers used for ChIP-qPCR.

**ID**	**Primer sequence(5′-3′)**
ChIP-qPCR-*PORC*-F	AAAACACACTCAATTATGATAGACCA
ChIP-qPCR-*PORC*-R	CGAGAAATTTTCGAGAACCAG
ChIP-qPCR-*PSY2*-F	GTAACCCAGCTGCCCACTT
ChIP-qPCR-*PSY2*-R	AAAGAATATGAGCATTATTGAGTCCA
